# COVID-19 Case Investigation and Contact Tracing Efforts from Health Departments — United States, June 25–July 24, 2020

**DOI:** 10.15585/mmwr.mm7003a3

**Published:** 2021-01-22

**Authors:** Kimberly D. Spencer, Christina L. Chung, Alison Stargel, Alvin Shultz, Phoebe G. Thorpe, Marion W. Carter, Melanie M. Taylor, Mary McFarlane, Dale Rose, Margaret A. Honein, Henry Walke

**Affiliations:** ^1^Division of Preparedness and Emerging Infections, National Center for Emerging and Zoonotic Infectious Diseases, CDC; ^2^Division of STD Prevention, National Center for HIV/AIDS, Viral Hepatitis, STD, and TB Prevention, CDC; ^3^CDC COVID-19 Response Team.

Case investigation and contact tracing are core public health tools used to interrupt transmission of pathogens, including SARS-CoV-2, the virus that causes coronavirus disease 2019 (COVID-19); timeliness is critical to effectiveness (*1*,*2*). In May 2020, CDC funded* 64 state, local, and territorial health departments^†^ to support COVID-19 response activities. As part of the monitoring process, case investigation and contact tracing metrics for June 25–July 24, 2020, were submitted to CDC by 62 health departments. Descriptive analyses of case investigation and contact tracing load, timeliness, and yield (i.e., the number of contacts elicited divided by the number of patients prioritized for interview) were performed. A median of 57% of patients were interviewed within 24 hours of report of the case to a health department (interquartile range [IQR] = 27%–82%); a median of 1.15 contacts were identified per patient prioritized for interview^§^ (IQR = 0.62–1.76), and a median of 55% of contacts were notified within 24 hours of identification by a patient (IQR = 32%–79%). With higher caseloads, the percentage of patients interviewed within 24 hours of case report was lower (Spearman coefficient = –0.68), and the number of contacts identified per patient prioritized for interview also decreased (Spearman coefficient = –0.60). The capacity to conduct timely contact tracing varied among health departments, largely driven by investigators’ caseloads. Incomplete identification of contacts affects the ability to reduce transmission of SARS-CoV-2. Enhanced staffing capacity and ability and improved community engagement could lead to more timely interviews and identification of more contacts.

During July 31–August 14, 2020, baseline data on four metrics for June 25–July 24, 2020 (the evaluation period) were submitted by 62 of 64 (97%) health departments funded through the Epidemiology and Laboratory Capacity for Prevention and Control of Emerging Infectious Diseases Cooperative Agreement (ELC)^¶^ to the Research Electronic Data Capture (REDCap) platform (*3*). These metrics, developed by the CDC COVID-19 Contact Tracing Innovations Support Team, were vetted by public health partners, including a number of ELC-funded health departments, and include the following: 1) average caseload per case investigator (the total number of probable and confirmed COVID-19 patients assigned for interview during the evaluation period divided by the total number of case investigators), average contact tracing load (the total number of contacts assigned for follow-up divided by the total number of contact tracers), and staffing model (separate, mostly separate, or the same health department staffing for case investigation and contact tracing); 2) case investigation timeliness (the percentage of persons with probable and confirmed COVID-19 prioritized for interview successfully reached within 24 hours by a health department staff member or representative); 3) contact tracing timeliness (the percentage of contacts notified of potential exposure to COVID-19 within 24 hours of elicitation of contact information by a patient); and 4) contact tracing yield, calculated as the number of contacts elicited divided by number of patients prioritized for interview. Because guidance for prioritization of patient interviews was not provided, health departments developed their own criteria, examples of which included interviewing patients when they became known to the health department or prioritizing patient interviews based on whether the patients were symptomatic, had underlying medical conditions, lived in congregate settings, or worked in health care occupations. Descriptive analyses of the four metrics were performed using SAS (version 9.4; SAS Institute). This activity was reviewed by CDC and was conducted consistent with applicable federal law and CDC policy.**

Among the 62 funded health departments, four (6.5%) (all U.S.-affiliated Pacific islands) reported no cases, and two (3.2%) submitted partial data and were excluded. Data from the remaining 56^††^ (90%) health departments were analyzed. Because completeness of reporting by health departments varied by metric, denominators varied. Health departments with incomplete data for a metric were excluded for that specific metric.

Among reporting health departments, the median caseload per investigator during the evaluation period was 31, ranging from one to 196, among 54 (96%) health departments with complete data for this metric (Table). Among patients prioritized for interview by these 54 health departments, a median of 57% were interviewed within 24 hours of report to the health department. Among 53 health departments that provided information on the average number of contacts assigned for follow-up per contact tracer, the median was 29, ranging from 0.5 to 200; a median of 55% of contacts were notified within 24 hours of elicitation by a patient. Among 48 health departments that reported information on contact notification, 27 (56%) reported that at least one half of contacts were notified within 24 hours of elicitation. However, 12 health departments reported that fewer than one third (<32%) of contacts were reached within 24 hours.

**TABLE:** COVID-19 case investigation and contact tracing metrics — 56 health departments, United States, June 25–July 24, 2020

Metric	Median* (IQR) [total range]	No. (%) of health departments
**Case investigation**		
Total cases assigned for interview during reporting period	8,306 (1,781–19,671) [22–280,033]	56 (100)
Average no. of cases assigned for interview per case investigator (caseload)	31 (15–68) [1–196]	54 (96)
Prioritized persons interviewed within 24 hrs	57 (27–82) [1–100]	54 (96)
**Contact tracing**		
Contacts elicited from cases during reporting period	7,498 (2,236–19,937) [124–95,775]	54 (96)
Average no. of contacts assigned for follow-up per contact tracer (contact tracing load)	29 (17–44) [0.5–200]	53 (95)
Contacts notified within 24 hrs of identification by a patient	55 (32–79) [4–100]	48 (86)

**Abbreviations:** COVID-19 = coronavirus disease 2019; IQR = interquartile range.

* Median caseload and contact tracing loads represent the median of the average per investigator or contact tracer.

Caseload and timeliness of case investigation were inversely correlated among 49 health departments with complete data for these metrics (Spearman correlation coefficient = –0.68) (Figure 1). Health departments with smaller average caseloads per investigator completed a larger proportion of patient interviews within 24 hours of report. Among four health departments that interviewed >90% of patients within 24 hours, investigators’ average caseloads were fewer than 30 patients each, whereas among four health departments with average caseloads >130 patients per investigator, <30% of interviews were completed within 24 hours.

**FIGURE 1:**
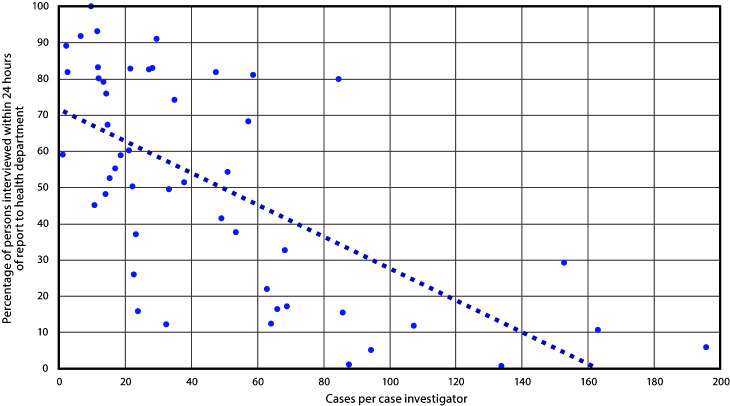
Association between COVID-19 caseload per health department investigator and timeliness of case interviews — 49 health departments, United States, June 25–July 24, 2020* **Abbreviation:** COVID-19 = coronavirus disease 2019. * The trendline represents the inverse correlation between the average caseload per case investigator and the timeliness of case investigations among 49 health departments.

When restricted to patients prioritized for interview (9,013), among 53 health departments that submitted complete data, 42 (79%) reported fewer than two contacts elicited per patient (median = 1.15). The number of contacts elicited per patient prioritized for interview was smaller in health departments with larger caseloads (Spearman correlation coefficient = –0.60) (Figure 2). These trends persisted in jurisdictions that allocated different staff members, mostly different staff members, or the same staff members to be case investigators and contact tracers (Spearman correlation coefficients = –0.89, –0.69, and –0.32, respectively).

**FIGURE 2:**
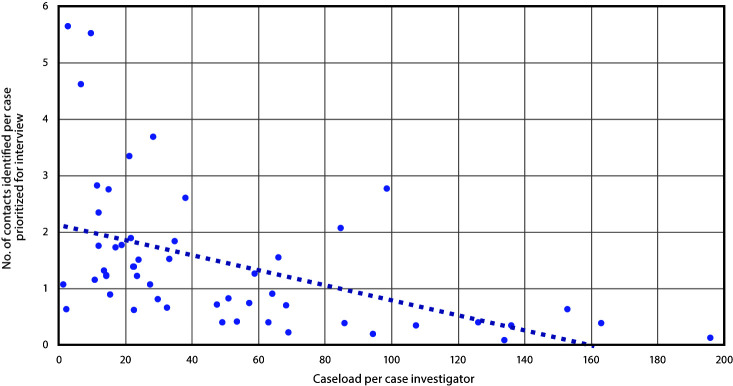
Association between the COVID-19 caseload per health department investigator and number of close contacts identified per case prioritized for interview — 52 health departments, United States, June 25–July 24, 2020* **Abbreviation:** COVID-19 = coronavirus disease 2019. * The trendline represents the inverse correlation between the average caseload per case investigator and the number of contacts elicited per patient prioritized for interview among 52 health departments.

## Discussion

Health departments’ capacity and ability to conduct timely and effective case investigation and contact tracing varied widely across the United States. The ideal workforce size to adequately conduct case investigation and contact tracing per jurisdiction^§§^ likely depends on several factors (*4*); however, the inverse relationship between staff member workload and completeness and timeliness of case investigation and contact tracing suggest that increases in staffing capacity might help reduce delays in interviewing patients and identify more contacts. Most state health departments are hiring more staff members to perform contact tracing^¶¶^ (*1*). Health departments might choose to prioritize case investigation and contact tracing based on whether persons are likely to be at higher risk for severe disease, live or work in congregate settings, or are part of a known cluster (*5*). Surges in cases might exceed the workforce capacity of jurisdictions to maintain high coverage of case investigation and contact tracing. Continued efforts to ensure notification of patients of their infection and contacts of their exposure are needed. CDC recommends use of prioritization measures to reach populations at risk as well as use of innovative technologies (*6*) to support this public health imperative.

Approximately one half of health departments were able to achieve a median interval of ≤24 hours from first notification of the patient to interview; likewise, approximately one half also were able to achieve a median interval of ≤24 hours from patient interview to contact notification, although these two groups did not always comprise the same health departments. These findings are comparable with those in recent reports that described median intervals of 1 day from patient report to interview and 1 and 3 days from case investigation to contact notification in two U.S. counties (*1*,*7*). The evaluation period in this report, June 25–July 24, 2020, corresponded to a time of increased COVID-19 incidence (*8*); the capacity of health departments in jurisdictions with large numbers of cases to conduct timely patient follow-up and contact notification could be overwhelmed.

The median number of contacts elicited per patient prioritized for interview was 1.15. The number of contacts elicited per patient would have been higher if limited to the number of patients who completed an interview rather than those who were prioritized for an interview; however, the number of patients who completed an interview was not collected at this time, and the calculation was not possible. A recent assessment of two North Carolina counties reported an average of 3.0 and 4.6 contacts named per interviewed patient during a similar time frame (*1*). A contact tracing team in central Pennsylvania identified 953 contacts elicited among 536 confirmed patients (1.8 contacts per patient) during March 24–May 28; the lower number of contacts per patient might be related to the widespread stay-at-home orders that were in effect during that time (*9*).

One contributor to low numbers of contacts elicited might be related to reluctance to engage in contact tracing efforts*** or to name persons other than household contacts (*1*). The number of contacts elicited might vary by caseload, owing to worker fatigue or inexperience; with higher caseloads, contact tracers might be less likely to persist with questioning to identify additional contacts.

The findings in this report are subject to at least four limitations. First, these data are self-reported by health departments and were likely generated from new data systems designed to monitor case investigation and contact tracing. New systems could be prone to errors and might not reflect complete performance within the jurisdiction. Second, data validity might be affected by health departments’ varying interpretations of definitions of metrics. These data include that obtained during health departments’ first reporting period on these metrics, which will continue to be refined. Third, these data precluded calculation of the average number of contacts elicited per patient who completed an interview, and therefore do not align with other studies’ methods of calculating contacts elicited (*1*); the actual number is likely higher, warranting cautious interpretation. Finally, an important component of contact tracing is laboratory test timeliness, which is not included in these data. During the COVID-19 pandemic, delays from the time of laboratory specimen collection to report to the health department can have substantial impact on total time to reach a contact (*2*,*9*); the absence of these data in an assessment of contact tracing timeliness is an especially important limitation of this report.

Delays in interviewing COVID-19 patients decrease the likelihood of quickly identifying and quarantining contacts. Low ascertainment of contacts affects the nation’s potential to interrupt the transmission of SARS-CoV-2 through rapid notification, quarantining, and testing. Caseloads within jurisdictions influence how quickly health departments can reach patients, which might influence the completeness of data used to reach contacts. Increasing staffing capacity might improve the timeliness of case interviews. Strengthening awareness regarding state and local health department contact tracing efforts might improve community perception or willingness to provide more complete lists of contacts.

**Summary**

**What is already known about this topic?**

Resources have been allocated to supplement the U.S. case investigation and contact tracing workforce as a public health tool to interrupt the spread of COVID-19.

**What is added by this report?**

Analysis of case investigation and contact tracing metric data reported by 56 U.S. health departments found wide variation in capacity and ability to conduct timely and effective contact tracing. Investigator caseload was inversely related to timely interviewing of patients and number of contacts identified per case.

**What are the implications for public health practice?**

Enhanced staffing capacity and ability and improved community engagement could lead to more timely contact tracing interviews and identification of more contacts.

## Footnotes

* https://www.cdc.gov/media/releases/2020/p0423-CARES-act.html; https://www.cdc.gov/media/releases/2020/p0518-hhs-funding-expand-testing-states.html.

† 50 U.S. states, Chicago, Houston, New York City, Philadelphia, Washington D.C., Los Angeles County, American Samoa, Northern Mariana Islands, Federated States of Micronesia, Guam, Palau, Puerto Rico, Marshall Islands, and U.S. Virgin Islands.

§ Patients prioritized for interview refer to persons with confirmed and probable COVID-19 reported to the health department in the official case report system. Prioritization of patients is defined differently by different health departments.

¶ https://www.cdc.gov/ncezid/dpei/epidemiology-laboratory-capacity.html.

** 45 C.F.R. part 46.102(l)(2), 21 C.F.R. part 56; 42 U.S.C. Sect. 241(d); 5 U.S.C. Sect. 552a; 44 U.S.C. Sect. 3501 et seq.

†† Alabama, Alaska, Arizona, Arkansas, California, Colorado, Connecticut, Delaware, Florida, Georgia, Hawaii, Idaho, Illinois, Indiana, Iowa, Kansas, Kentucky, Louisiana, Maine, Maryland, Massachusetts, Michigan, Minnesota, Mississippi, Montana, Nebraska, Nevada, New Hampshire, New Jersey, New Mexico, New York, North Carolina, North Dakota, Oregon, Pennsylvania, Rhode Island, South Carolina, South Dakota, Tennessee, Texas, Utah, Vermont, Virginia, Washington, West Virginia, Wisconsin, Wyoming, Chicago, Houston, New York City, Philadelphia, Los Angeles County, Northern Mariana Islands, Guam, Puerto Rico, and U.S. Virgin Islands

§§ https://www.gwhwi.org/estimator-613404.html.

¶¶
https://www.npr.org/sections/health-shots/2020/06/18/879787448/as-states-reopen-do-they-have-the-workforce-they-need-to-stop-coronavirus-outbre .

*** https://www.scientificamerican.com/article/contact-tracing-a-key-way-to-slow-covid-19-is-badly-underused-by-the-u-s/.
